# Resting-state fMRI activation is associated with parent-reported phenotypic features of autism in early adolescence

**DOI:** 10.3389/frcha.2024.1481957

**Published:** 2024-11-05

**Authors:** Robert Hickson, Liberty Hebron, Eva M. Muller-Oehring, Anastasia Cheu, Andres Hernandez, Orsolya Kiss, Marie Gombert-Labedens, Fiona C. Baker, Tilman Schulte

**Affiliations:** ^1^ Department of Psychology, Palo Alto University, Palo Alto, CA, United States; ^2^ Neuroscience Program, SRI International, Menlo Park, CA, United States; ^3^ School of Medicine, Stanford University, Stanford, CA, United States

**Keywords:** rs-fMRI (resting state fMRI), default mode network (DMN), features of autism spectrum, preadolescence, neuroactivation

## Abstract

**Introduction:**

Autism Spectrum Disorder (ASD) is characterized by deficits in social cognition, self-referential processing, and restricted repetitive behaviors. Despite the established clinical symptoms and neurofunctional alterations in ASD, definitive biomarkers for ASD features during neurodevelopment remain unknown. In this study, we aimed to explore if activation in brain regions of the default mode network (DMN), specifically the medial prefrontal cortex (MPC), posterior cingulate cortex (PCC), superior temporal sulcus (STS), inferior frontal gyrus (IFG), angular gyrus (AG), and the temporoparietal junction (TPJ), during resting-state functional magnetic resonance imaging (rs-fMRI) is associated with possible phenotypic features of autism (PPFA) in a large, diverse youth cohort.

**Methods:**

We used cross-sectional parent-reported PPFA data and youth rs-fMRI brain data as part of the two-year follow-up of the Adolescent Brain Cognitive Development (ABCD) study. Our sample consisted of 7,106 (53% male) adolescents aged 10-13. We conducted confirmatory factor analyses (CFAs) to establish the viability of our latent measurements: features of autism and regional brain activation. Structural regression analyses were used to investigate the associations between the six brain regions and the PPFA.

**Results:**

We found that activation in the MPC (*β* = .16, *p* < .05) and the STS (*β* = .08, *p* < .05), and being male (*β* = .13, *p* < .05), was positively associated with PPFA. In contrast, activation in the IFG (*β* = −.08, *p* < .05) was negatively associated.

**Discussion:**

Our findings suggest that regions of the “social brain” are associated with PPFA during early adolescence. Future research should characterize the developmental trajectory of social brain regions in relation to features of ASD, specifically brain regions known to mature relatively later during development.

## 1 Introduction

Autism spectrum disorder (ASD) is associated with deficits in social cognitive processes and self-referential processes (1, 2). In addition to social impairment, ASD is clinically associated with restricted, repetitive behaviors and interests (3). ASD symptoms can be observed at a sub-clinical threshold, and it is established that a broad autism phenotype exists, which captures a range of social functioning on a continuum (4).

Neurofunctional substrates of ASD have been characterized by atypical brain activation (5) and altered functional connectivity (FC) among brain regions (6) during resting-state functional magnetic resonance imaging (rs-fMRI). Studies consistently highlight abnormalities in several key brain networks associated with social cognition, communication, and sensory processing. For instance, reduced connectivity within the default mode network (DMN), including regions such as the medial prefrontal cortex (MPC) and posterior cingulate cortex (PCC), has been linked to social deficits in ASD (7, 8). Similarly, atypical functioning of the salience network (SN), particularly in regions like the anterior insula and anterior cingulate cortex (ACC), has been associated with challenges in processing socially relevant stimuli (9). Other studies have pointed to hyper- or hypoconnectivity in the amygdala, a region critical for emotion regulation, as being involved in ASD-related emotion processing difficulties (10).

Despite this body of evidence, ASD does not have a single, agreed-upon biomarker or identifiable neural structure that characterizes the condition (11). It is also unclear how features of autism are commonly represented during neurodevelopment. This lack of a definitive biomarker has prompted further investigation into more specific neural processes, such as self-referential processes associated with the DMN, which is activated during rs-fMRI (12, 13). Poor self-referential processing in ASD is linked to poor theory of mind, a critical social cognitive process (2). DMN activation in brain regions associated with self-referential processing may provide insight into mechanisms associated with features of autism during development in adolescents who do not have an ASD diagnosis but show subclinical ASD symptoms. We aim to build upon existing research looking at select neural correlates of the DMN related to self-referencing and theory of mind in ASD (14) by examining youth during early adolescence, a critical period of self-identity development and social development, when social cognition and self-reflection may be most indicated.

Brain structures of interest in the present study include the MPC, PCC, superior temporal sulcus (STS), inferior frontal gyrus (IFG), angular gyrus (AG), and the temporoparietal junction (TPJ). These brain regions of the DMN have neurofunctional overlap with the regions of what some have termed the theory of mind network [ToMN; (15, 16)], a functional network involved in social cognition (17, 18). These brain structures can be categorized based on functional purpose in the context of thinking about and interacting with the social world and are collectively considered regions of the “social brain” (15, 19). The MPC is linked with mentalizing or theory of mind (i.e., mentalizing or reflecting on the emotional/mental states of self and others), person perception, self-perception, and self-referential thinking (2, 20, 21). The temporal regions contribute to conceptual and semantic knowledge related to mentalizing (22). The PCC has been theorized as a sub-component for self-referential and other social cognitive processes (20, 23). The STS and TPJ are linked with predicting biological movement, assignment of agency to self and others (i.e., theory of mind and self-other distinction), perspective taking, and empathy (15, 24). The shared functions of these brain regions as they relate to features of autism, specifically during adolescent development, are not yet fully understood. Maturation of brain regions is not uniform during adolescence, with frontal brain regions maturing later (25, 26) and less neurodevelopmental change seen in ASD (27) influencing social development and broad autism phenotype during adolescence (28).

In this study, we examined rs-fMRI activation in regions that are known to be involved in social cognition, especially those social cognitive processes related to phenotypic features of autism. Brain activation during rs-fMRI is known to be heterogeneous (29) and changes throughout development (30). Accordingly, we tested whether intrinsic activity in brain regions of interest are associated with possible phenotypic features of autism (PPFA) in a large, diverse youth sample across the United States.

## 2 Method

### 2.1 Participants

We analyzed cross-sectional data from the two-year follow-up (2YFU) visit from the Adolescent Brain Cognitive Development (ABCD) study (5.1 data release). The data included in the study is available on the NIMH Data Archive via data access request. The study used resting-state functional magnetic resonance imaging (rs-fMRI) data and parent report data of 7,108 adolescents between the ages of 10–13 (*M* = 11.95, SD = 0.65). Table 1 describes demographic characteristics of the sample. Our analysis’ inclusion and exclusion criteria were consistent with the criteria for participation in the ABCD study (31). Accordingly, children with a confirmed diagnosis of moderate or severe ASD were excluded from ABCD study enrollment at baseline. Although the ABCD study does not have data on clinically diagnosed ASD, it is possible that youth with mild ASD and ASD feature endorsement are included. Additionally, our sample was limited to participants with brain imaging data collected at their 2YFU visit, thus does not include all participants actively enrolled in the ABCD study. The 2YFU time point was used because it was the only time point that had data for our variables of interest in the 5.1 data release.

**Table 1:** Participant demographics.

	*N* (%)
Age (mean, SD)	11.95, 0.65
Race	
White	3,954 (55.63%)
Hispanic	1,371 (19.29%)
Black/African American	919 (12.93%)
Asian	138 (1.94%)
Other	726 (10.21%)
Sex assigned at birth	
Male	3,749 (52.74%)
Female	3,359 (47.26%)
Gender identity	
Male	3,742 (52.64%)
Female	3,353 (47.17%)
Trans male	1 (0.01%)
Trans female	3 (0.04%)
Gender queer	2 (0.03%)
Different [than provided options]	4 (0.06%)
Refused to answer	1 (0.01%)
Don't know	2 (0.03%)

### 2.2 Measures

#### 2.2.1 Brain regions

We used the temporal variability of 22 cortical parcellations from the Destrieux brain atlas (32) during rs-fMRI. Temporal variability describes fluctuations in the BOLD (blood-oxygen-level-dependent) signal over a specified time course (i.e., the length of the rs-fMRI scan) from which neural activity in the region is inferred (33–35). The cortical parcellations used for analysis were grouped to form brain regions of interest: MPC, PCC, STS, IFG, AG, and TPJ. The Destrieux atlas’ anatomical parcellation names (index numbers) used for each region are as follows:

MPC: The left and right anterior part of the cingulate gyrus and sulcus (6, 80).
  The left and right middle-anterior part of the cingulate gyrus and sulcus (7, 81).
PPC: The left and right posterior part of the cingulate gyrus and sulcus (8, 82).
  The left and right posterior-dorsal part of the cingulate gyrus (9, 83).
  The left and right posterior-ventral part of the cingulate gyrus (10, 84).
STS: The left and right STS (73, 147).
IFG: The left and right opercular part of the inferior frontal gyrus (12, 86).
  The left and right orbital part of the inferior frontal gyrus (13, 87).
  The left and right triangular part of the inferior frontal gyrus (14, 88).
AG: The left and right AG (25, 99).
TPJ: The left and right supramarginal gyrus (26, 100).

#### 2.2.2 Phenotypic features of autism

The study uses three parent-reported questions with 5-point scales ranging from “Not at all” to “Nearly every day” related to PPFA taken from Module #18 (autism spectrum disorders) of the computerized *Kiddie Schedule for Affective Disorders and Schizophrenia for DSM-5* (KSADS-COMP) (36). The questions include:

(1) “In the past two weeks, how often has your child had trouble maintaining eye contact and looking at you or other people when they are talking with your child?”
(2) “In the past two weeks, how often have you worked real hard to keep routines and activities the same so your child would not get upset?”
(3) “In the past two weeks, how often did your child do unusual body movements like hand flapping, head weaving, body rocking, or body spinning?”

### 2.3 Data analysis

We tested our hypothesis using confirmatory factor analysis (CFA) and structural equation modeling with the lavaan package in R (37). CFA for fMRI data has been an established approach with several advantages compared to data reduction techniques such as principal components analysis and partial least squares (38). We estimated all models using diagonally weighted least squares because of its appropriate use for ordinal data that do not meet the assumptions of non-normality (39), which is true of the features of autism data used in our study. We evaluated model fit using chi-square (*χ*^2^), Comparative Fit Index (CFI; values > .90 suggest acceptable fit and values > .95 suggest good fit), Tucker-Lewis Index (TLI; values > .90 suggest acceptable fit and values > .95 suggest good fit), standardized root mean residual (SRMR; values < .08 suggest good fit), root mean square error approximation (RMSEA; values < .08 suggest good fit).

Initially, we conducted CFAs in order to establish the viability of our latent measurements of PPFA as reported by parents and brain regions of interest, specifically the MPC, PCC, STS, IFG, AG, and the TPJ. We used the 22 parcellations from the Destrieux atlas as observed indicators for the six-factor CFA for the brain regions of the MPC, PCC, STS, IFG, AG, and TPJ. For our primary analysis, we regressed the latent factor of features of autism onto the six latent factors of the brain regions of interest and sex assigned at birth.

## 3 Results

### 3.1 Confirmatory factor analysis of PPFA

**Figure 1:**
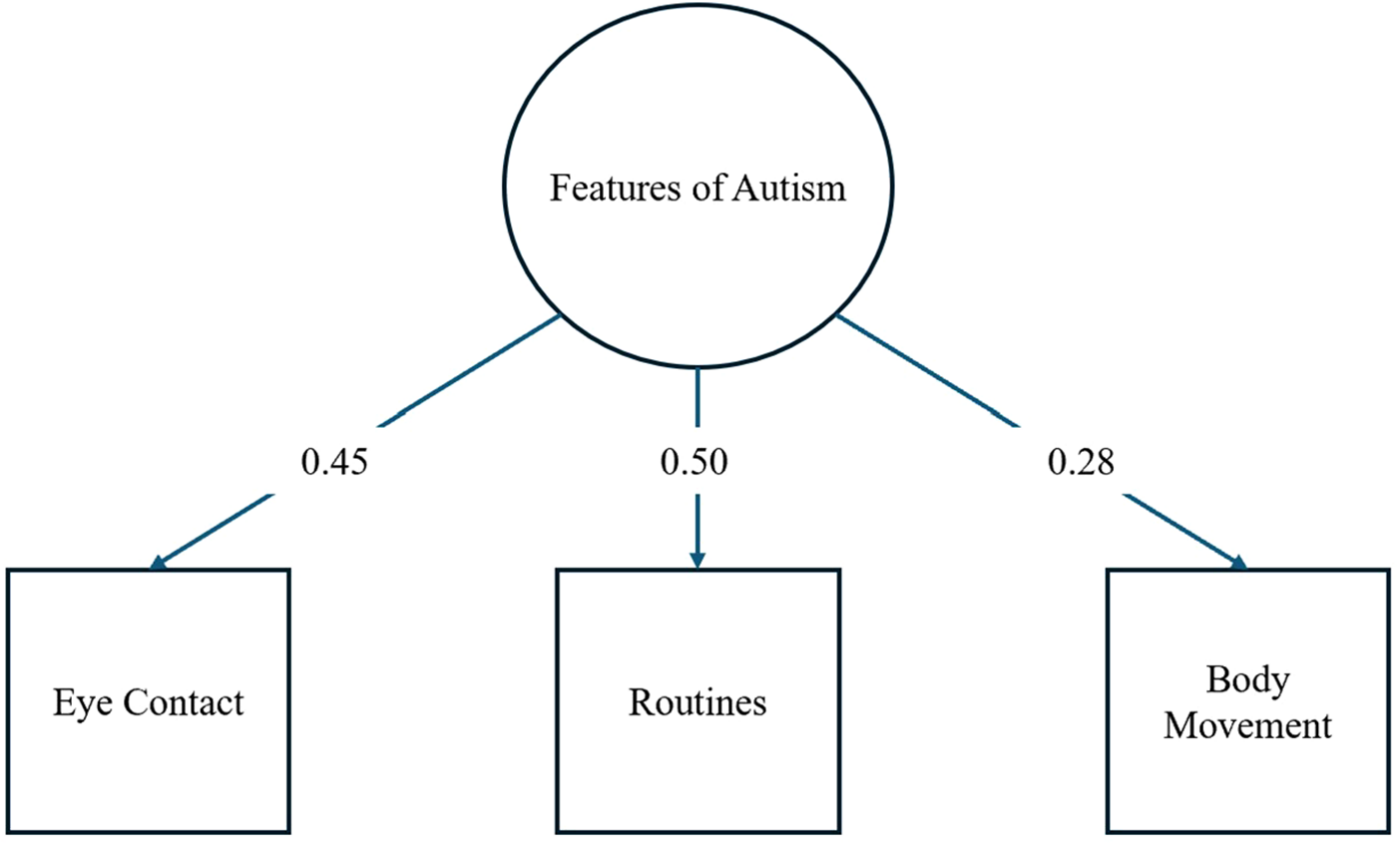
Model for one factor CFA for PPFA with factor loadings.

Figure 1 shows the results of the CFA for PPFA suggesting adequate model fit (CFI = 1.00, TLI = 1.00, SRMR = 0.00, RMSEA = 0.00, *χ*^2^ < 0.001) with loadings ranging from .28 to.50 (see Figure 1). Thus, the three parent-report questions from the K-SADS questionnaire appropriately form a latent construct characterized as PPFA.

### 3.2 Confirmatory factor analysis for brain regions of interest

**Figure 2:**
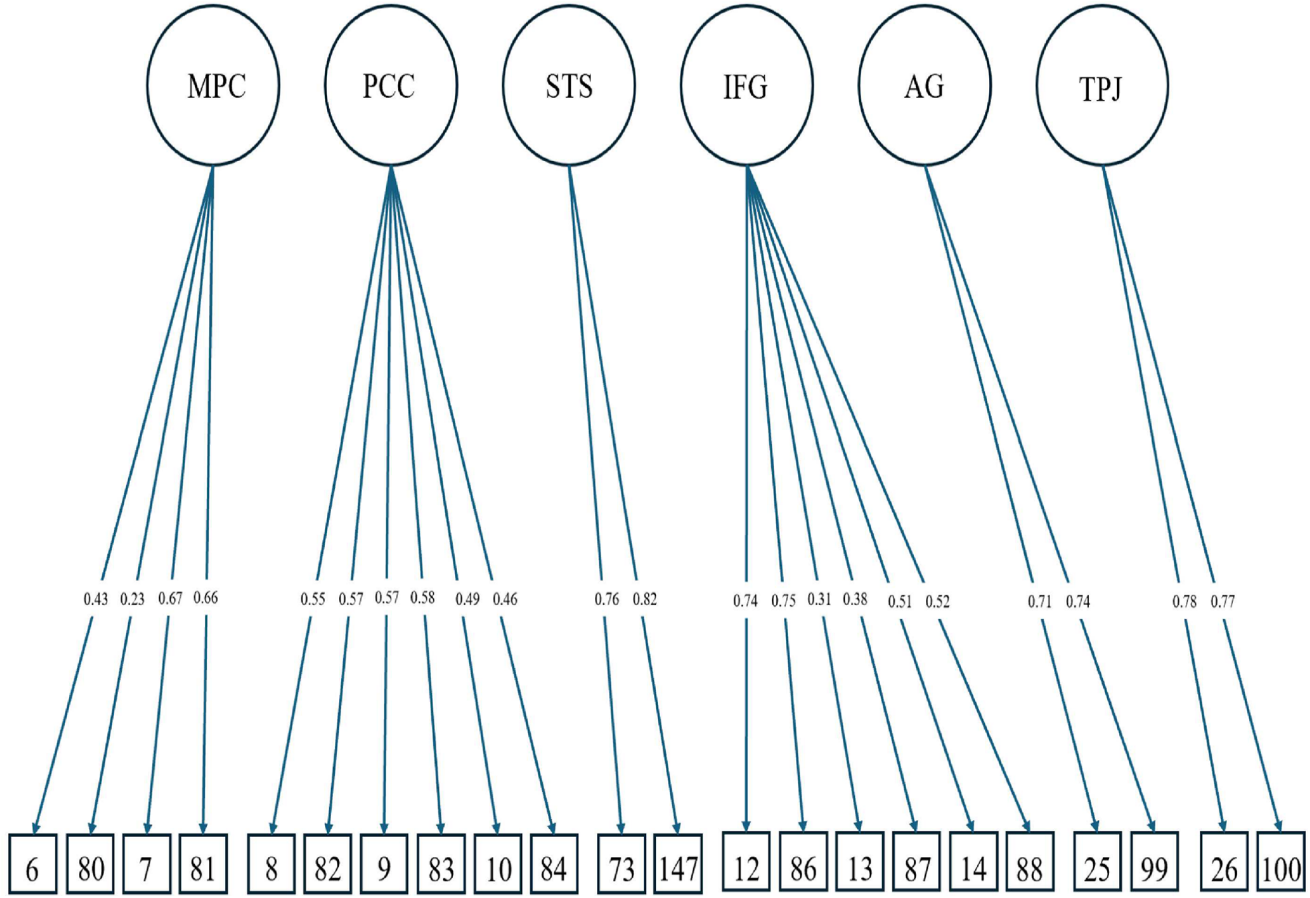
Model for six factor CFA for brain regions of interest with destrieux atlas cortical parcellations as the observed indicators. See Table 2 for correlations among latent factors. The parcellations numbers correspond with the Destrieux atlas’ index number as follows: the left and right anterior part of the cingulate gyrus and sulcus (6, 80), the left and right middle-anterior part of the cingulate gyrus and sulcus (7, 81), the left and right posterior part of the cingulate gyrus and sulcus (8, 82), the left and right posterior-dorsal part of the cingulate gyrus (9, 83), the left and right posterior-ventral part of the cingulate gyrus (10, 84), the left and right STS (73, 147), the left and right opercular part of the inferior frontal gyrus (12, 86), the left and right orbital part of the inferior frontal gyrus (13, 87), the left and right triangular part of the inferior frontal gyrus (14, 88), the left and right AG (25, 99), the left and right supramarginal gyrus (26, 100).

Figure 2 shows the CFA for the brain regions of interest converging with an adequate model fit (CFI = .97, TLI = .96, SRMR = .08, RMSEA = .03, *χ*^2^ < .001), thus indicating the six brain regions of interest for our analysis: MPC, PCC, STS, IFG, AG, and TPJ. Correlations between the targeted brain regions ranged from moderate to large and were all positive in direction (see Table 2).

**Table 2:** Correlation between and psychometric information for all predictor variables.

	1	2	3	4	5	6
1. Medial prefrontal cortex (MPC)	(.66)					
2. Posterior cingulate cortex (PCC)	.77	(.74)				
3. Superior temporal sulcus (STS)	.55	.68	(.77)			
4. Inferior frontal gyrus (IFG)	.73	.57	.43	(.78)		
5. Angular gyrus (AG)	.58	.71	.60	.50	(.69)	
6. Temporal parietal junction (TPJ)	.64	.69	.53	.55	.69	(.75)
7. Sex assigned at birth	.10	.14	.12	.05	.13	.15

Cronbach's alpha on diagonal for continuous variables. Point-biserial correlations were calculated for sex assigned at birth (0 being male and 1 being female) and brain regions. Therefore, positive values indicate a greater correlation between the neurofunctional activity and being assigned female at birth.

### 3.3 Regression analysis of selected brain regions and sex assigned at birth predicting PPFA

**Figure 3:**
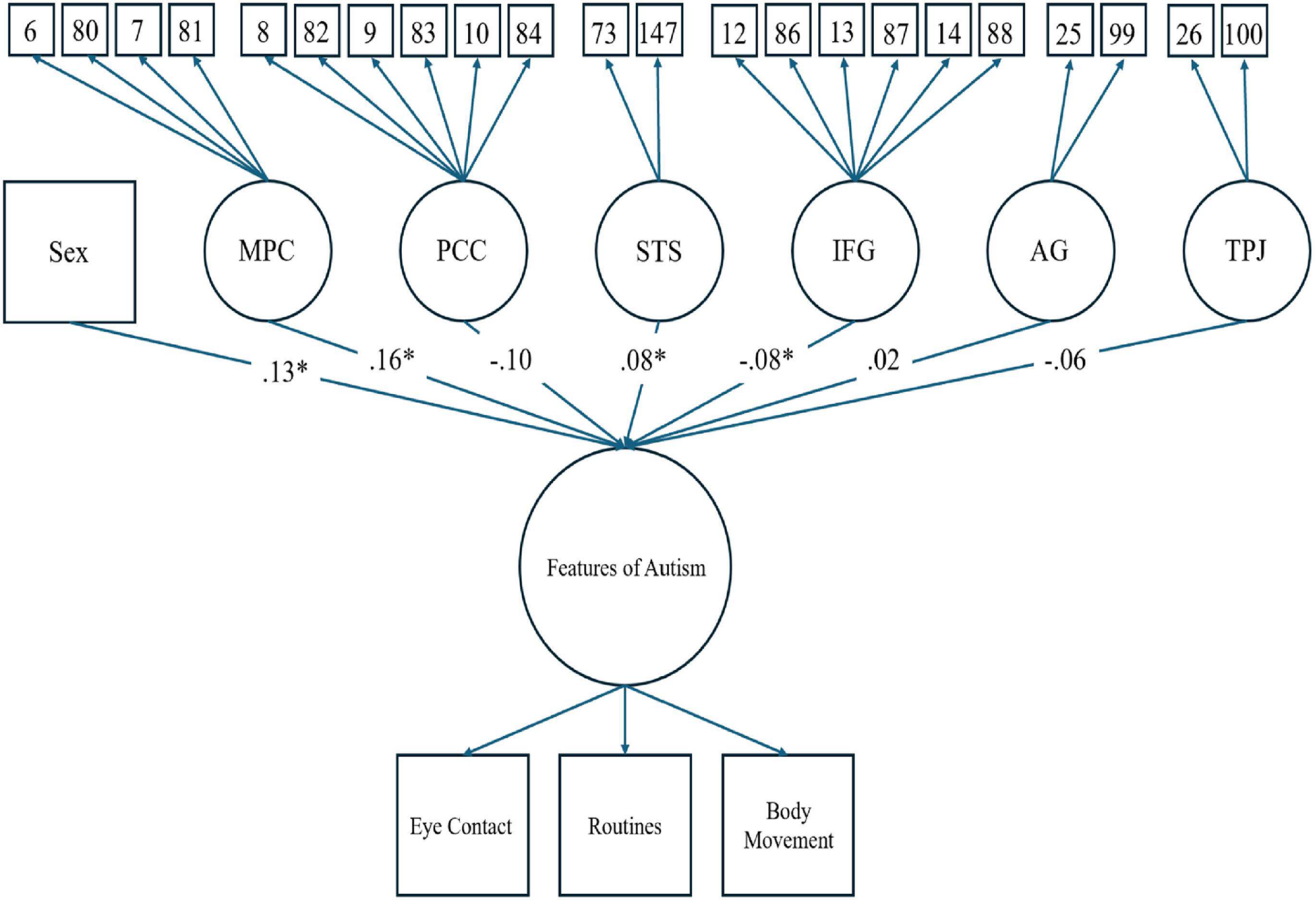
Full structural regression model (measurement and structural) for a latent factor of PPFA predicted by sex assigned at birth and six latent factors of brain regions of interest. Regression loadings from the six brain regions and sex assigned at birth are listed. An asterisk indicates a significant relationship at the .05 level. Loading values for the latent factor of features of autism and the six brain regions are identical to those from the preliminary CFAs (see Figures 1, 2).

**Figure 4:**
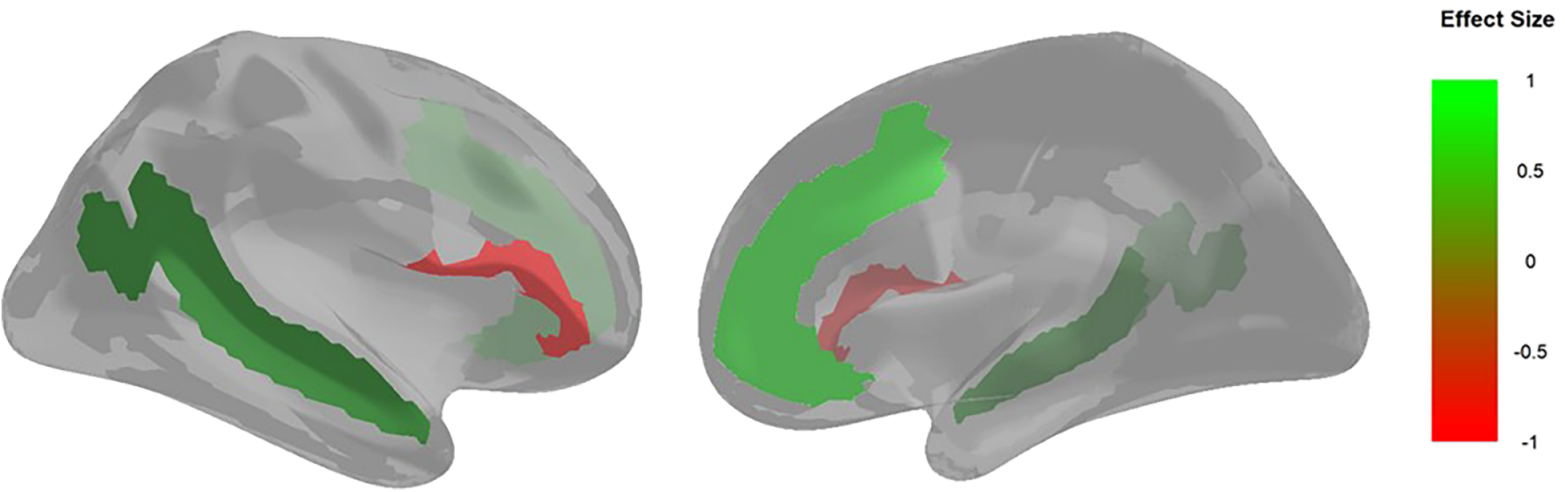
Brain visualization of areas with activation that are significantly associated with PPFA. The image on the left is a lateral view of the right hemisphere, and the image on the right is a medial view of the right hemisphere. The colors represent the standardized regression loadings from the structural regression model which are significant at the .05 level. Areas with a positive effect size (i.e., greater resting-state activation associated with PPFA) are green. Areas with a negative effect size (i.e., less resting-state activation associated with PPFA) are red.

Figure 3 shows the results of the structural regression model, which converged with adequate fit (CFI = .94, TLI = .93, SRMR = .03, RMSEA = .07, *χ*^2^ < .001). The structural regression of a latent factor of PPFA regressed onto brain regions interest and sex assigned at birth showed activation in the MPC (*β* = .17, *p* < .05) and the STS (*β* = .08, *p* < .05) to be positively associated with PPFA. Additionally, being assigned male at birth was positively associated with PPFA (*β* = .13, *p* < .01). Conversely, activation in the IFG (*β* = −.08, *p* < .05) showed to be negatively associated with PPFA. There was not a significant association between the PCC (*β* = −.11, *p* = .13), AG (*β* = .02, *p* = .71), and TPJ (*β* = −.05, *p* = .19) with parent-reported PPFA in our youth cohort (see Figures 3, 4). Point-biserial correlations between sex assigned at birth and neurofunctional activity of the six brain regions showed that female participants have greater activation in all identified regions, and the difference was small in magnitude, ranging from .05 to .15 (see Table 2). The above reported PPFA–activation relationships in MPC, STS, and IFG regions were significant after controlling for neurofunctional differences between males and females.

## 4 Discussion

Our findings suggest that resting-state neurofunctional activity is indicative of PPFA in early adolescence. We found that greater resting-state activation in the MPC and the STS is associated with increased PPFA as reported by parents. These findings are concordant with literature that relates the MPC and the STS to self-referential thinking (20, 40), a cognitive process consistent with resting-state activity. Activation in areas related to self-referential thinking have been linked to a heightened focus on internal cues [i.e., thoughts and perceptions; (41)]. Difficulties in distinguishing between one's own thoughts and perspectives and those of others is a hallmark of autism that contributes to social challenges (42, 43). Furthermore, we found that less resting-state activation in the IFG is associated with increased PPFA. The IFG has been implicated in emotion recognition and regulation (44). Interestingly, there is evidence of IFG hypoactivation in early adolescence (around age 12) in youth with ASD, but no differences in IFG activation in adults with ASD compared to group-matched control adults (45). In this context, our finding of hypoactivation in the IFG being associated with greater PPFA in early adolescence is consistent with the literature (46–48) and builds upon previous findings as we find IFG hypoactivation during resting state in youth at ages 10–13 years.

Additionally, we find that anterior brain regions (MPC, STS, IFG) have a greater association with PPFA compared to posterior regions (PCC, AG, TPJ). This is in line with the posterior to anterior brain maturation pattern occurring during early adolescence through adulthood (28) and is consistent with altered fronto-posterior brain connectivity being associated with autistic features/traits (49). Thus, as frontal brain regions continue to mature, there is potential for a reduction in phenotypic features of autism observed in youth during early adolescence. We also found that being assigned male at birth was also associated with greater PPFA. This is consistent with sex differences in puberty onset (50) and brain maturation (51), with males showing later onset and maturation compared to females. Notably, this finding could also reflect differences between the presentation of features of autism, with females having displaying greater behavior camouflaging (52), thus less likely to be noticed and reported on the parent-report measure. Altogether, our results provide novel evidence that neural activation in DMN brain structures, which align with regions of the ToMN, during resting state are associated with PPFA in early adolescence.

Our study focuses on a cohort of preadolescents without a clinical diagnosis of ASD at baseline who express a range of parent-reported PPFA at the second-year follow-up. Therefore, our results infer how neurofunctional activity in regions of the DMN relates to PPFA among a diverse cohort of preadolescents, where PPFA was based on three questions, which cannot capture the full complexity and heterogeneity of ASD. Additionally, although the symptoms-measure scale was intended to capture features of ASD, the specific features (i.e., eye contact, maintaining a routine, and unusual body movement) can occur in conjunction with other neurodevelopmental disorders or psychopathologies, such as attention-deficit/hyperactivity disorder, anxiety, or depression. Therefore, these transdiagnostic features should not be understood as exclusive to ASD, but rather as indicators of neurodevelopment that is consistent with ASD features. Future research should aim to identify and disambiguate contributory factors that manifest as both distinct resting-state neurofunctional activity and transdiagnostic features of atypical psychosocial development. Furthermore, our study's focus on three questions related to PPFA limits the scope of how neurofunctional differences in the DMN impact functioning. Our findings should be extended with research investigating how differences in DMN activity relate to domains of functioning in adolescence, such as social engagement, academic performance, and emotion regulation.

Beyond limitations related to the measurement of features of autism, the interpretation of our study results is limited due to being cross-sectional. It is not understood if greater resting-state activation in the MPC and STS and less activation in the IFG can predict features of autism throughout adolescence and young adulthood. Longitudinal studies are warranted to characterize the trajectories of these examined brain regions, specifically of brain regions known to mature relatively later in development. Considering that alteration in the DMN has also been associated with social anxiety and other mental health disorders (53, 54), future research is needed to explore if intrinsic social brain activation pattern associated with PPFA during early adolescence in youth without a diagnosis of ASD is a risk factor for developing socioemotional mental health issues, such as social anxiety, during late adolescence and young adulthood. Overall, a better understanding of the neurofunctional substrates of features of autism will provide a better understanding of its heterogeneity, developmental timing, and targeted treatment needs.

## Data Availability

The data analyzed in this study is subject to the following licenses/restrictions: A data use agreement is needed to access the ABCD data and sharing of raw data to those without access is not allowed. Requests to access these datasets should be directed to https://nda.nih.gov/abcd.
